# SDF-1 Promotes Endochondral Bone Repair during Fracture Healing at the Traumatic Brain Injury Condition

**DOI:** 10.1371/journal.pone.0054077

**Published:** 2013-01-22

**Authors:** Xiaoqi Liu, Changlong Zhou, Yanjing Li, Ye Ji, Gongping Xu, Xintao Wang, Jinglong Yan

**Affiliations:** Department of Orthopedic Surgery, The First Affiliated Hospital, Harbin Medical University, Harbin, P.R. China; University of Pittsburgh, United States of America

## Abstract

**Purposes:**

The objective of this study was to investigate the role of stromal cell-derived factor-1 (SDF-1) and its receptor, CXCR4, on bone healing and whether SDF-1 contributes to accelerating bone repair in traumatic brain injury (TBI)/fracture model.

**Materials and Methods:**

Real-time polymerase chain reaction and immunohistochemical analysis were used to detect the expression of SDF-1 during the repair of femoral bone in TBI/fracture model. The TBI/fracture model was treated with anti–SDF-1 neutralizing antibody or AMD3100, an antagonist for CXCR4, and evaluated by histomorphometry. In vitro and in vivo migration assays were used to evaluate the functional effect of SDF-1 on primary mesenchymal stem cells.

**Results:**

The expression of SDF1 and CXCR4 messenger RNA was increased during the bone healing in TBI/fracture model but was less increased in fracture only model. High expression of SDF-1 protein was observed in the surrounding tissue of the damaged bone. Treated with anti–SDF-1 antibody or AMD3100 could inhibit new bone formation. SDF-1 increased mesenchymal stem cell chemotaxis in vitro in a dose-dependent manner. The in vivo migration study demonstrated that mesenchymal stem cells recruited by SDF-1 participate in endochondral bone repair.

**Conclusion:**

The SDF-1/CXCR4 axis plays a crucial role in the accelerating fracture healing under the condition of TBI and contributes to endochondral bone repair.

## Introduction

In clinical practice, patients with sustained traumatic brain injury (TBI) display accelerated fracture healing [1] and overgrowth of callus and ectopic ossification is even observed in the muscle [2], but the mechanisms involved in these events remain unclear. In recent years, researchers have investigated the pathophysiologic mechanisms underlying these osteogenic phenomena in patients with TBI, and the explanation for these events is probably multifactorial [3]–[10]. Some researchers have focused on the influence of different levels of nerve injuries. Hara-Irie et a1. found that in sciatic innervation-losing rats, the cementing line of the trabecula in the growth plate was evidently increased in the late stage, suggesting that the osteoclastic activity at the epiphysis was regulated to some extent by the regulation of calcitonin gene-related polypeptide positive nerve fiber [11]. Olfinowski believed that the cerebral cortical neuron has a two-way regulatory action on osteogenesis and that the hyperactivity of neurons at the spinal level stimulates osteogenesis [12].

Other studies have recognized that the expression changes in growth factors also influence the speed of bone healing. Wildburger et al. showed that the levels of basic fibroblast growth factor (b-FGF) undergo a 3-fold rise after a fracture, and in head injured patients with an associated fracture this level has been shown to rise by up to seven times that of normal. The amounts of growth factors or cytokines in the blood, which regulate osteogenesis or stimulate the release of local growth factors, are notably increased after brain injury, leading to an overgrowth of callus and acceleration of bone healing [13]. Bidner et al. showed that patients with TBI possess a humoral mechanism for enhanced fracture-healing and that the serum of patients with brain injuries was able to promote the osteoblastic mitosis and multiplication in rats in a dose dependent manner [14]. However, the effect of chemokines on fracture healing in a TBI model has yet to been proven. We are specifically interested in stromal cell derived factor 1 (SDF-1), since many prior publications have shown that SDF-1 is critical to hematopoietic stem cell (HSC) and possibly MSC migration, and SDF-1 can be used to target stem cells to a desired site within the body [15]. Coincidentally, SDF-1 is also involved in the recruitment of inflammatory cells and other types of stem cells, including tissue-committed stem cells [16]. Recent studies have demonstrated that the chemokine receptor CXCR4 together with SDF-1 forms an important axis determining the retention and/or migration of stem cells, either from the bone marrow to the injury site or vice versa. High levels of binding of CXCR4 to SDF-1 at the injury site ensure the retention of mobilized CXCR4-positive cells to the repair site [17]. Moreover, SDF-1 is induced in the periosteum of injured bone, and it promotes endochondral bone repair by recruiting MSCs to the site of injury [14]. Furthermore, a previous report demonstrated that SDF-1 is important for migration of marrow stromal cells to bone marrow and that this migration occurs in a dose-dependent manner [15].

Based on these observations, we hypothesized that SDF-1 would play an important role in endochondral bone repair in femoral fracture model under TBI condition. Our results lead to a further understanding of the physiologic mechanisms underlying accelerating fracture healing and suggested new strategies for the therapeutic use of SDF-1 to promote successful bone healing.

## Materials and Methods

### Murine Models

Forty-three, 6-week-old, C57BL/6 mice were used in this study. All animals received humane care in accordance with “The Code of Ethics of the World Medical Association” for animal experiments (Revision of Directive 86/609/EEC), and this study was conducted with the permission of the ethics committee of Harbin Medical University”. The animals were divided into two groups: the fracture-only group (20 animals), in which only the fracture was created; the TBI/fracture group (23 animals) in which TBI was produced with an impact acceleration system [18], [19] besides the fracture. Similar to that observed in patients with TBI, a reproducibly impart a diffuse axonal injury was shown in this model [20].

### Femoral Fracture Model

Femoral osteotomy and fixation were produced as described by Bonnarens et al [21]. Briefly, the left rear leg was shaved, swabbed with povidone-iodine for disinfection, and draped. A transverse osteotomy was made at the mid-shaft of the femur, and intramedullary fixation was performed using a stainless steel wire (diameter, 1.5 mm). The fracture was produced and stabilized. The wires were cut on the surface of the intercondylar groove. The operative site was closed with sutures. Unrestricted activity was allowed after recovery from anesthesia. When the animals were killed, the fractured limb was disarticulated at the hip joint.

### TBI/Fracture Model

TBI was created with use of an established model, as previously reported [18], [19]. After the animals were anaesthetized with isoflurane, the skull was exposed and a craniectomy was performed laterally, between the central suture and the left temporal ridge. The animal’s head was then positioned on a foam block under 1.5 m of polyvinyl chloride tubing that was attached to a metal stand. A nickel-plated cap was fitted on the exposed dura. The opening of the tubing was centered over the nickel plate directly above the animal’s head. A 20-g weight was then dropped from the top of the tubing, from a height of exactly 30 cm. Once impact was made, the foam block and the animal were moved away from the tubing to avoid a rebound impact, thus limiting the injury to a single impaction. The scalp was closed without replacement of the bone flap. The creation of the femoral shaft fracture was similar with femoral fracture model. Anesthesia was discontinued, and the animal was assessed for exclusion criteria (latency of pinna, corneal reflexes, and righting response). The total mortality of the TBI/fracture and fracture groups combined was 18.2%. Mice that died during surgery were replaced and an additional procedure was performed. Animals were returned to their cages and allowed to recover under observation.

Postoperatively, four randomly chosen animals from each group were killed at 2 days post-injury; whole brain specimens were harvested for histologic analysis and documentation of brain injury. Specimens were prepared and midcoronal sections were cut and stained with hematoxylin and eosin, as previously described [18], [19], in order to validate the extent of the head injury.

### Real-time Polymerase Chain Reaction Analysis

At 1, 2, 3, and 7 days post surgery, the mice were killed and the femoral bone and surrounding tissue were obtained. Mouse tissue samples were snap-frozen in liquid nitrogen and were homogenized with Polytron (Kinematica, Tokyo, Japan), and mRNA was extracted using TRIzol (Gibco BRL, Carlsbad, CA, USA) and purified using an RNeasy Mini Kit (Qiagen, Valencia, CA, USA). The RNA was reverse transcribed to cDNA and amplified via polymerase chain reaction (PCR). The amplified products were subjected to electrophoresis in 2% agarose gels. The measurements of gene expression were used for baseline calculations using RotorGene 3000 Software (Corbett Research, Mortlake, Australia) according to the manufacturer’s instructions. All gene expression data were normalized against GAPDH.

### Bone Marrow Stromal Cell Culture

Primary mouse bone marrow–derived stromal cells (BMSCs), harvested as previously described [22], were counted and subsequently plated in cell culture flasks in Dulbecco modified Eagle medium (DMEM; Gibco-BRL, Grand Island, NY, USA) supplemented with 20% fetal bovine serum (FBS; Gibco-BRL) and 100 mg/ml penicillin–streptomycin (Gibco-BRL) [23]. Non-adherent cells were removed from the cultures after 2 days via a series of phosphate buffered saline (PBS) washes and subsequent medium changes. Adherent cells were expanded as monolayer cultures under standard culturing conditions (37°C, 5% CO_2_) with medium changes every 3 days. At 80–90% confluence, cells were harvested with trypsin/EDTA (Gibco BRL) and replated by splitting them (usually 1∶3) at a density of 50–60%, which was regarded as post-passage 1. These handlings were repeated several times until a sufficient number of cells were produced. The cell surface antigens were analyzed by flow cytometry (data not shown). Bromodeoxyuridine (10 µM; BrdU Cell Proliferation Kit; Amersham, Piscataway, NJ, USA) was used to label mouse BMSCs that were incubated in culture medium for 24 hours before the cells were harvested. And about 92.3±1.4% (mean ± SD) of BrdU-labeled mouse BMSCs were BrdU positive by immunocytochemical assay (data not shown).

### In vitro chemotaxis Assay

In vitro migration assays were carried out using the 96-well ChemoTx Disposable Chemotaxis System (Neuroprobe, Gaithersburg, MD, USA) with an 8-µm pore membrane, as previously described [24]. Briefly, 1×10^5^ mouse BMSCs in α -MEM containing 0.1% bovine serum albumin were put on the top of the system, and the bottom chamber contained rMuSDF-1 (PeproTech, Rocky Hill, NJ, USA) at different concentrations (0, 10, and 100 ng/ml) in 500 µl of medium. rMuSDF-1 (100 ng/ml) and AMD3100 (1 µM or 10 µM), an antagonist of CXCR4, were applied to the upper chambers for the chemokinesis and inhibition assays. 100 ng/ml of pertussis toxin (Alexis, Biomol, USA) was added to the chambers used as a control. After 24 hours of incubation, H&E stained to visualize and quantify cell migration to the lower side of the membrane.

### Loss-of-function Studies *in vivo*

To further investigate the influence of SDF-1 on bone healing, loss-of-function studies were performed in femoral fracture model under TBI condition. Mice received mouse anti–SDF-1 neutralizing antibodies (clone79014, R&D Systems, Minneapolis, MN, USA) or AMD3100. A total of 84 µg anti–SDF-1 neutralizing antibody diluted in PBS was injected intraperitoneally on days 2, 4, 7, and 10 after surgery (336 µg total). The mice were killed on day 14 for histologic analysis of new bone formation. AMD3100 (100 µg total) was administrated continuously, as described above, and the mice were killed on day 7.

### In vivo Chemotaxis Assay

After the surgery, the BrdU-labeled mouse BMSCs were collected in PBS at a cell concentration of 1.0×10^6^/ml, and a 200 µl aliquot of this cell suspension was injected into each mouse through the tail vein in TBI/fracture model. The distribution of the labeled cells around the bone was evaluated. To further assess the inhibitory effect of the receptor antagonist, an Alzet micro-osmotic pump (model 1002; Durect, Cupertino, CA, USA) was used to continuously deliver AMD3100 (5 mg/kg/day; Sigma-Aldrich, St. Louis, MO, USA) dissolved in PBS to the site of fracture subcutaneously as described previously [25]. The mice were killed on day 7 for histomorphometric analysis. Immunostaining was performed to mark the migrated BrdU-positive cells, and the number of migrated cells was counted. Cell counting was performed by three blinded observers, and the mean ratio of BrdU-positive chondrocytes to total chondrocytes in the formed soft callus was calculated.

### Histological and Histomorphometric Analyses

At the time that the animals were killed, the femora were fixed for one day in 4% paraformaldehyde and phosphate-buffered saline solution. After fixation, the bones were rinsed and decalcification was carried out with 14% EDTA (pH 7.2–7.4) for 3 to 4 weeks on a shaker at 4°C (the EDTA solution was changed once per week). The intramedullary pins were removed from the bones before embedding and sectioning. With use of a scalpel, the femora were divided into two halves by identifying the center of the callus and making a cross-sectional cut (the fracture center was determined with the aid of the radiographs that had been made during the fracture procedure); another two cuts were placed 5 mm proximal and distal to the center of the fracture. The resulting two specimens of bone represented the entire fracture callus. After the specimens were processed with a thickness of 5–7 µm and stained with hematoxylin and eosin. Three nonconsecutive sections were used for histomophometric analyses. Images of histologic sections were captured using a digital camera connected to a light microscope with an original magnification of 50×. And measurements were carried out directly on the digital images at a magnification of 50×. Commercial software (Adobe Photoshop CS3) was used to count the number of pixels.

The borders of the newly formed bone were manually marked on the computer screen using a digital pen. Subsequently, the pixels within this marked area were counted using computer software as previously described [20].

For immunohistochemical analysis, deparaffinized sections were washed in PBS with 0.1% Triton X-100 (Sigma-Aldrich) and endogenous peroxidase was blocked using a solution of 30% methanol and 3% H_2_O_2_ for 30 minutes. The tissue sections were pretreated with 10% normal rabbit serum diluted in PBS (blocking buffer) for 30 minutes. The primary antibody was a goat anti–SDF-1 polyclonal antibody (Santa Cruz Biotechnology, Santa Cruz, CA, USA), which was diluted 1∶200 in blocking buffer. Sections were incubated in diluted primary antibody for 24 hours at room temperature and then washed three times with PBS containing 0.1% Triton X-100 for 5 minutes each time. The secondary antibody used was a biotinylated rat anti-goat polyclonal antibody (R&D Systems, Minneapolis, MN, USA) diluted 1∶1000 in blocking buffer. After three washes, the tissue sections were incubated in diluted secondary antibody for 30 minutes at room temperature and then washed three times in PBS with 0.1% Triton X-100 for 5 minutes each time. The reaction products were visualized using a Vectastain ABC and DAB Peroxidase Substrate Kits (Vector, Burlingame, CA, USA), according to the manufacturer’s instructions. Negative controls were stained as above but without primary antibody. Immunohistochemical detection of BrdU-labeled cells was performed using a Cell Proliferation Kit (Amersham, Piscataway, NJ, USA) according to the manufacturer’s instructions.

### X-ray Imaging

Bone healing was monitored by X-rays taken at 2 weeks post-grafting. An X-ray film (40 kV and 2.0 mA with a constant X-ray to object to film distance of 100 cm) was used to determine the status of bone formation.

### Statistical Analysis

Data were analyzed using SPSS for Windows (version 13.0; SPSS, Chicago, IL, USA). Data are presented as the mean ± SD and were analyzed with the Student’s *t*-test. A *P*-value of less than 0.05 was considered statistically significant.

## Results

In our experience, no signs of infection were found in either group. All animals in the brain-injury group showed signs of substantial neurologic impairment at the time of injury; these signs consisted of flexed posturing of the forelimbs as well as spastic extension of the hindlimbs and tail, as has been previously observed by other authors [18], [19]. Both groups were able to walk after the injury, and the groups showed no differences in the ability to walk by one day after the injury.

### The Volume of Callus was Minimal by the Inhibition of SDF-1

**Figure 1:**
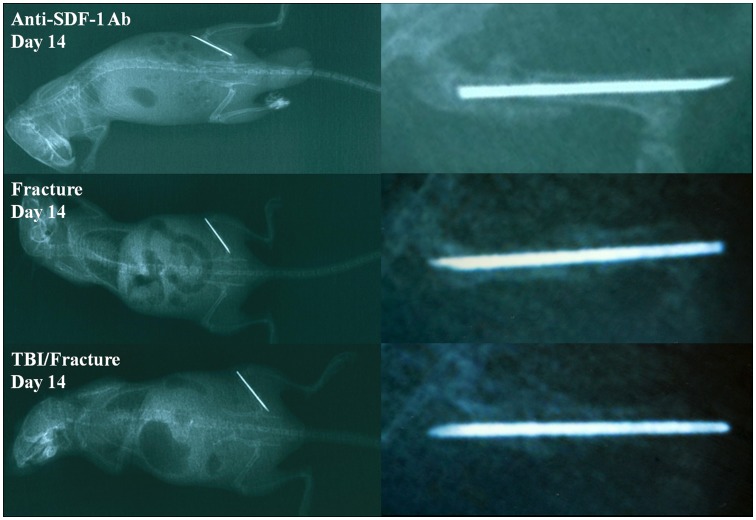
X-ray radiographs of fractured femur in fracture and TBI/fracture groups. At 14 days after fracture, the fracture line was visible in all groups. The callus became dense and callus formation at the fracture site in TBI/fracture group occurred more rapidly than those of the fracture group, whereas callus formation was minimal by the inhibition of SDF-1.

At day 14 after operation, X-ray radiographs showed that the evidence of callus formation was found at the fracture site in both the TBI/fracture group and the fracture group, whereas callus formation was minimal by the inhibition of SDF-1. The volume of callus in TBI/fracture group was larger than that in the fracture group and the callus formation occurred more rapidly at the fracture site in TBI/fracture model (Figure 1).

### High Expression of the SDF-1 Protein in the TBI/Fracture Model

**Figure 2:**
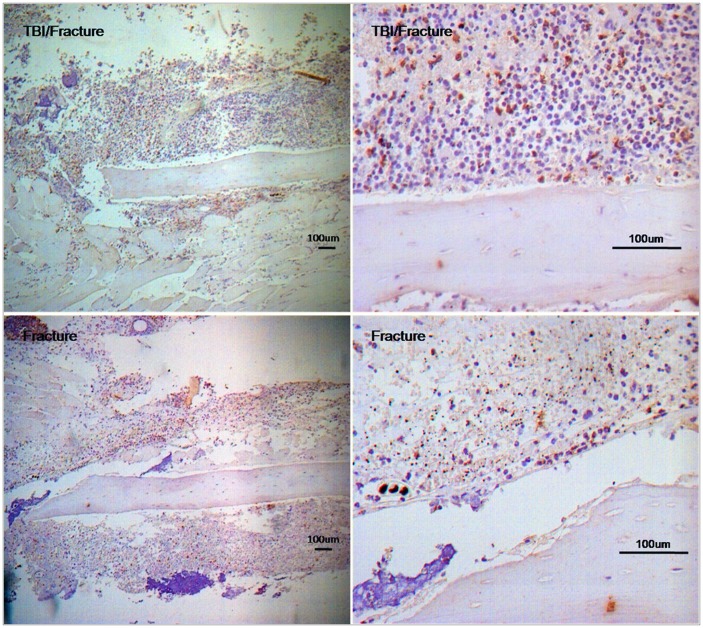
Immunohistochemical staining for SDF-1 in groups TBI/fracture and fracture only on day 2 after surgery. The expression of SDF-1 was observed in both groups. High expression of SDF-1 protein was detected at the surrounding tissue of the damaged bone in the TBI/fracture group.

Immunohistochemical analysis was performed to examine the localization of the SDF-1 protein expressed during the acute phase of bone healing. The expression of SDF-1 was observed in both groups at the growth plate and endosteum (results not shown), as previously reported [26]. High expression of SDF-1 protein was detected at the surrounding tissue of the damaged bone on day 2 in the TBI/fracture group (Figure 2, top). In contrast, less protein expression was observed in the fracture group (Figure 2, bottom). These results collectively demonstrated that an increase in SDF-1 in TBI/fracture than fracture only may be one of the mechanisms of accelerating bone repair in bone fracture healing with TBI during the acute phase of structural bone healing.

### High Expression Levels of SDF-1 and CXCR4 mRNAs in the TBI/Fracture Model

**Figure 3:**
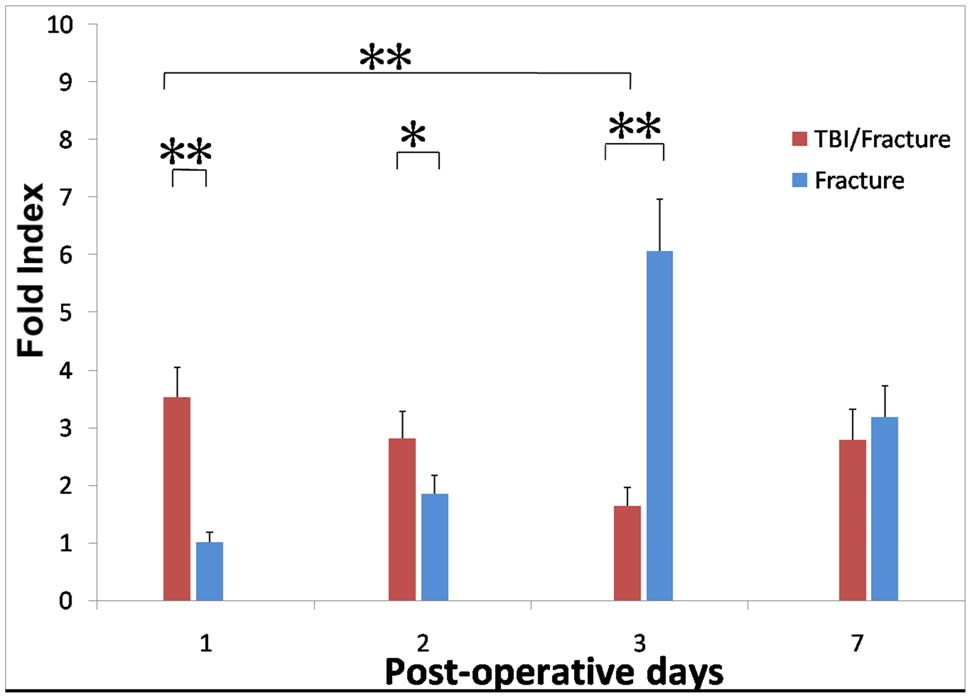
Time course of *SDF1* mRNA expression in TBI/fracture and fracture only models, as analyzed by real-time quantitative polymerase chain reaction (PCR) (n = 4 at each time point). SDF-1 mRNA expression increased by 3.5-fold in the TBI/fracture group on day 1 when compared with that in fracture group (*P*<0.001), and 1.5-fold higher in the TBI/fracture group than that in the fracture group on day 2. This expression was almost similar to the fracture group on day 7. Expression levels are the fold index versus the day 1 level in fracture only model. Values are the mean and SD results from triplicate real-time PCR analyses (*P<0.05; **P<0.001).

**Figure 4:**
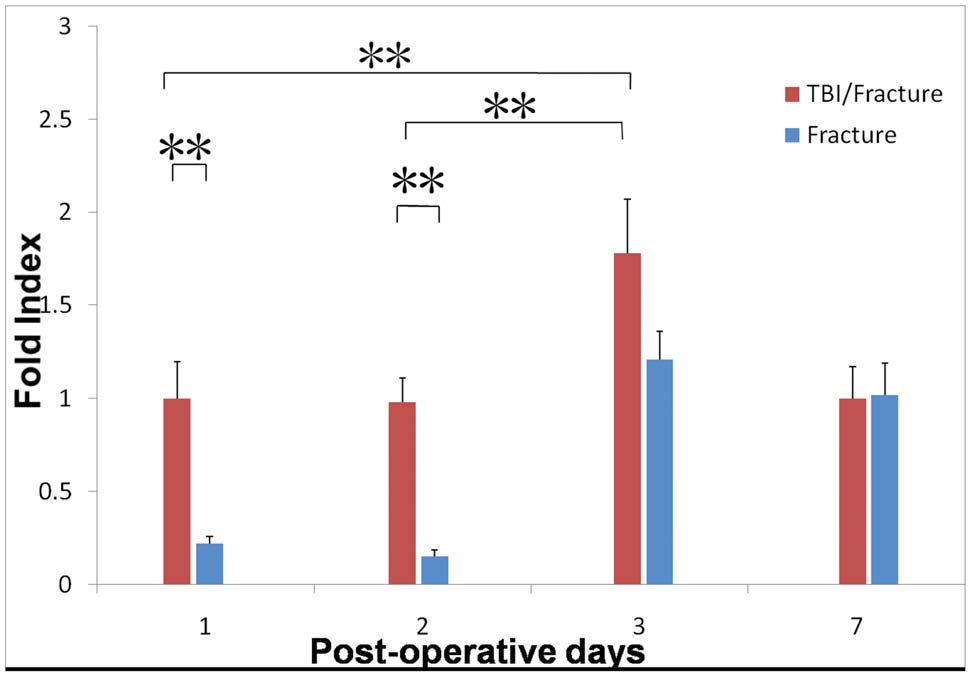
Time course of *CXCR4* mRNA expression in TBI/fracture and fracture only models, as analyzed by real-time quantitative polymerase chain reaction (PCR) (n = 4 at each time point). CXCR4 mRNA expression increased by 4.8-fold on day 1 in the TBI/fracture group compared with that in the fracture group (*P*<0.001), and further increased by 6-fold on day 2 (*P*<0.001) and nearly 1.5-fold on day 3 (*P*<0.001). Expression levels are the fold index versus the day 1 level in TBI/fracture only model. Values are the mean and SD results from triplicate real-time PCR analyses (*P<0.05; **P<0.001).

Both the TBI/fracture and fracture groups were used to investigate the involvement of SDF-1 and CXCR4 in the acute phase of skeletal repair. Total RNA was extracted from the site of the femur injury on days 1, 2, 3, and 7 (n = 4 for each time point), and real-time PCR was used to analyze the expression levels of the SDF-1 and CXCR4 mRNA. SDF-1 mRNA expression increased by 3.5-fold in the surrounding tissue of the damaged bone in the TBI/fracture group on day 1 (*P*<0.001) when compared with that in fracture group, and SDF-1 mRNA expression was 1.5-fold higher in the TBI/fracture group than that in the fracture group on day 2 (*P = *0.014; Figure 3). This expression was decreased by 3-fold on day 3 and was almost similar to the fracture group on day 7. At the same time, CXCR4 mRNA expression increased by 4.8-fold on day 1 in the TBI/fracture group compared with that in the fracture group (*P*<0.001). CXCR4 expression further increased by 6-fold on day 2 (*P*<0.001) and nearly 1.5-fold on day 3 (*P*<0.001; Figure 4). And the data of Q-PCR was shown in Table 1. These results indicated the involvement of SDF-1 and CXCR4 in the acute phase of endochondral bone healing.

**Table 1:** Effects of SDF-1 on stem cell recruitment in vivo and in vitro.

Group	Day after surgery	GAPDH	SDF-1	CXCR4	SDF-1/GAPDH	CXCR4/GAPDH
TBI/Fracture	1	2.13E−01	2.90E−04	6.89E−04	1.36E−03	3.23E−03
	2	1.67E−01	1.79E−04	3.85E−04	1.07E−03	2.31E−03
	3	1.68E−01	1.11E−04	2.89E−03	6.61E−04	1.72E−02
	7	2.72E−01	2.94E−04	3.89E−03	1.08E−03	1.43E−02
Fracture						
	1	3.06E−01	1.17E−04	4.46E−03	3.82E−04	1.46E−02
	2	3.14E−01	2.18E−04	4.42E−03	6.94E−04	1.41E−02
	3	3.16E−01	7.27E−04	8.25E−03	2.30E−03	2.61E−02
	7	2.35E−01	2.85E−04	3.41E−03	1.21E−03	1.45E−02

### Effects of SDF-1 on Stem Cell Recruitment in vivo and in vitro

**Figure 5:**
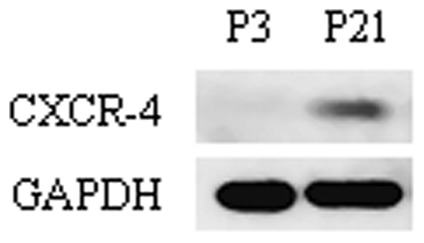
The expression levels of CXCR4 protein were analysed by Western blot. The band of CXCR4 protein was obviously weaker in cells at passage 3 than those of cells at 21passages in culture.

**Figure 6:**
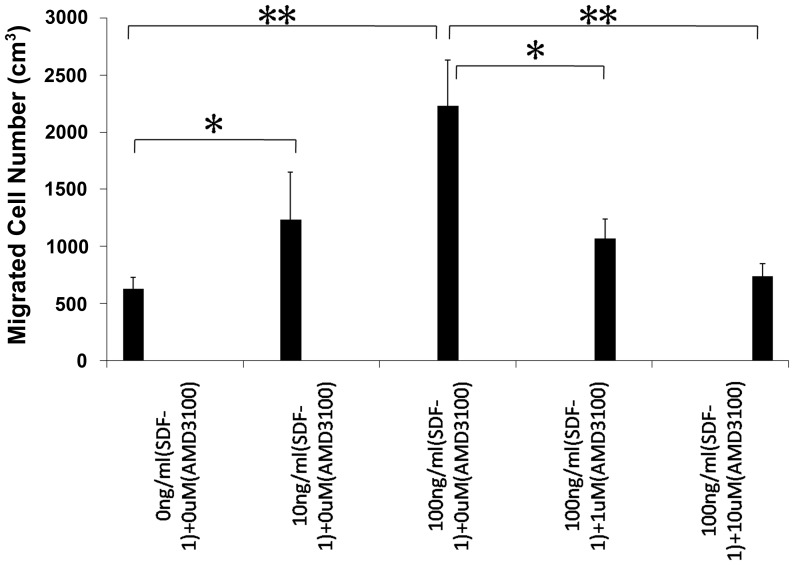
In vitro chemotaxis assay. 10 ng/ml rMuSDF-1 induced the migration of mouse BMSCs by 96%, and 100 ng/ml rMuSDF-1 by 254%. The effect was reduced after treated with AMD3100. Values are the mean and SD results from 4 independent experiments (*P<0.05; **P<0.001).

It has been previously reported that SDF-1 can mediate the mobilization and migration of bone marrow-derived stem and progenitor cells to the sites of organ injury [27]–[32]. Thus, we performed in vitro and in vivo cell migration assays using primary mouse BMSCs to verify our hypothesis that SDF-1 works as a chemoattractant of MSCs to bone-healing sites during endochondral bone repair. Firstly, flow cytometry was used to evaluate the phenotypic characterization of mouse BMSCs. In our study, mouse BMSCs became homogeneously negative for CD34 and CD45 at post-passage 21, which was consistent with a previous report [33]. Moreover, mouse BMSCs at post-passage 21 expressed higher level of CXCR4 protein than that in post-passage 3 (Figure 5), confirming the proficient reaction of these cells to SDF-1. Based on these results, mouse BMSCs from post-passage 21 to post-passage 25 were used for all subsequent assays. We used a chemotactic chamber assay to examine the in vitro chemotactic effect of SDF-1 on mouse BMSCs. 10 ng/ml rMuSDF-1 induced the migration of mouse BMSCs by 96%, and 100 ng/ml rMuSDF-1 by 254% (Figure 6). Treatment with 1 µM AMD3100, an antagonist of CXCR4, reduced the effect by 52% and 10µM AMD3100 by 67% (Figure 6). These results suggested that CXCR4 mediated the migration induced by SDF-1. Taken together, the results demonstrated the functional effect of the SDF-1/CXCR4 axis on the in vitro migration of mouse BMSCs.

**Figure 7:**
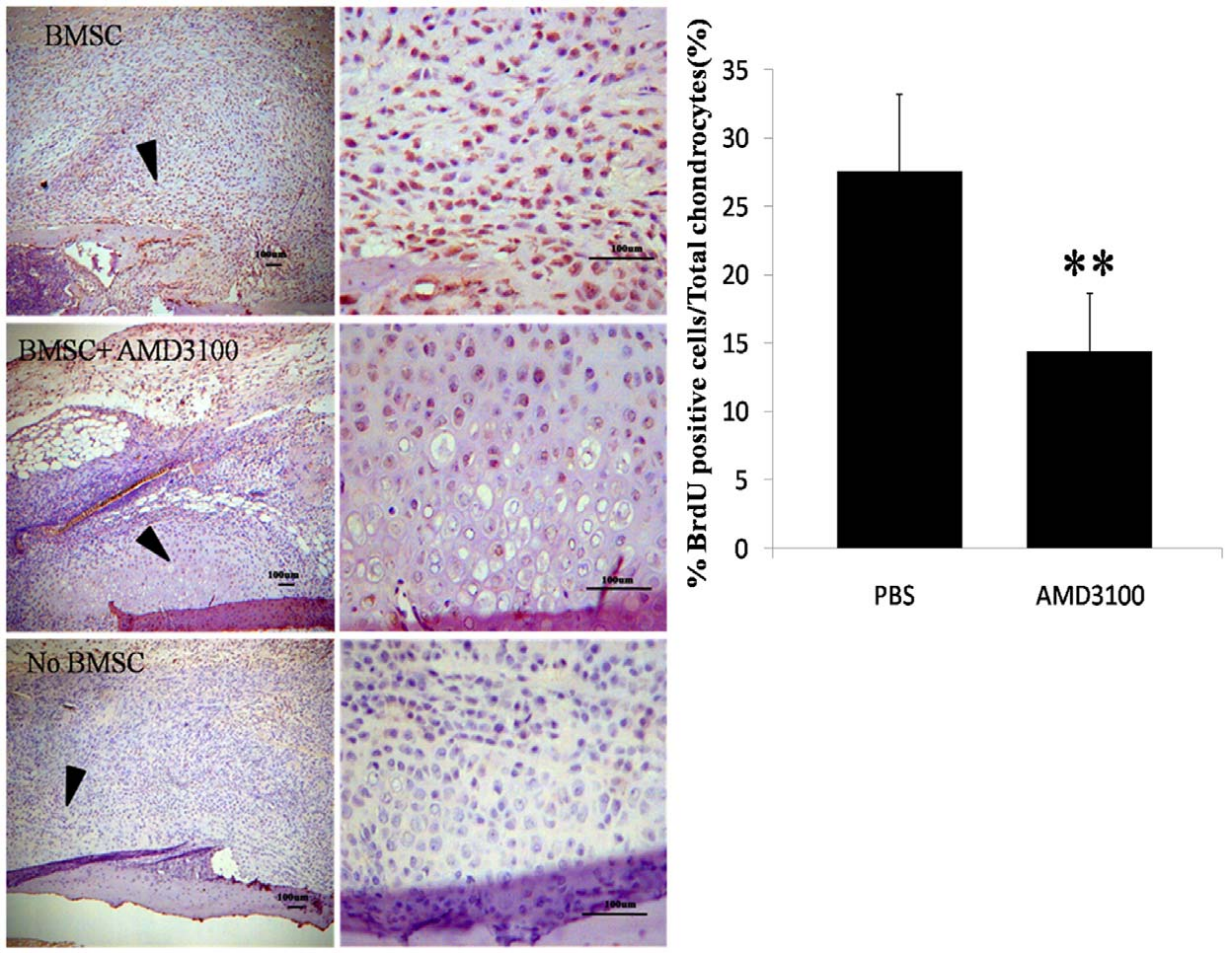
In vivo chemotaxis assay in TBI/fracture model. Sections obtained on day 7 after surgery for histologic and immunohistochemical analysis. In the specimens treated with PBS, positive BrdU staining was detected in the developing callus. The number of migrated cells was significantly reduced when treated with AMD3100 (**P<0.001).

Next, in vivo migration assay was performed in TBI/fracture model to investigate whether SDF-1 could recruit MSCs toward the sites of bone repair. 2×10^5^ BrdU-labeled mouse BMSCs were transplanted intravenously into the mice after surgery, and immunohistochemical analysis was performed to evaluate the migration of these cells to sites around the damaged bone on day 7. AMD3100 or PBS was used for the inhibition assays. Positive BrdU staining was detected in the developing callus formed around the damaged bone in the specimens treated with PBS, suggesting migration of transplanted mouse BMSCs to the damaged bone (Figure 7, left, top). In contrast, the number of migrated cells was significantly reduced when treated with AMD3100 (Figure 7, left, middle), indicating that the SDF-1/CXCR4 axis indeed regulate the migration of mouse BMSCs *in vivo*. Furthermore, we observed that 27.6% of total chondrocytes in the fracture callus were BrdU positive in PBS-treated specimens, while 14.4% were BrdU positive in AMD3100-treated specimens (Figure 7, right). These results demonstrated that the SDF- 1/CXCR4 axis had a crucial role during recruitment of MSCs toward the fracture sites during the early phase of bone repair *in vivo*.

### Inhibition of SDF-1 Resulted in Reduced New Bone Formation

**Figure 8:**
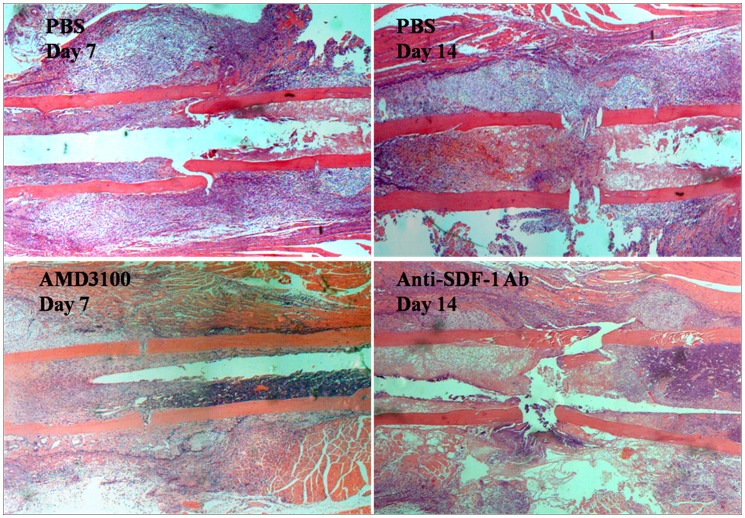
Loss-of-function study. New bone formation was significantly reduced at the fracture sites of the anti–SDF-1 antibody- and AMD3100-treated mice in comparison with the specimens treated with PBS on days 14 and 7, respectively. Stained with hematoxylin and eosin.

Loss-of-function study was performed to evaluate the effect of SDF-1 on successful bone repair using two different reagents, an anti–SDF-1 neutralizing antibody and AMD3100. After surgery, a series of intraperitoneal injections of anti–SDF-1 neutralizing antibodies or continuous administration of AMD3100 was performed on the mice in TBI/fracture model, and the mice were killed for histologic analysis on days 14 and 7, respectively. New bone formation was significantly reduced by 69% and 47% at the fracture sites of the anti–SDF-1 antibody- and AMD3100-treated mice in comparison with the specimens treated with PBS, respectively, suggesting that SDF-1 played a critical role in normal bone repair (Figure 8).

## Discussion

This study indicated that SDF-1 played an important role in endochondral bone repair in our TBI model. Firstly, determining the expressing pattern of SDF-1 was crucial for understanding its role during accelerated bone healing. So we performed gene expression and immunohistochemical analysis and observed that the surrounding tissue of the long bones indeed expressed the SDF-1 mRNA. The expression of the SDF-1 and CXCR4 mRNAs was significantly up-regulated in the TBI/fracture group during the acute phase of bone repair, whereas a lower increase was detected in the fracture only group. It was previously reported that SDF-1 is expressed at the endosteum and the growth plate of normal long bones in adults [26], but a recent study showed that SDF-1 is expressed at the periosteum during embryonic endochondral bone development and that expression is substantially reduced after birth [34]. Our observations indicated that there was no apparent SDF-1 expression at those locations. Another important issue for understanding the role of SDF-1 during accelerated bone repair is its regulated expression during the repair process. Increased expression of the SDF-1 mRNA was observed on days 1 and 2 in our model, although other studies have demonstrated an increase in SDF-1 expression within 24 hours after injury [29], [31], [32]. It was previously reported that SDF-1 is regulated by a hypoxia-specific transcriptional factor, hypoxia-inducible factor 1 (HIF-1), and that the expression of SDF-1 may increase rapidly after loss of blood supply [32]. However, the function of trophic vasculature would be affected after TBI and subsequent hypoxia may have resulted in a relative increase in SDF-1 expression in the TBI/fracture group. Furthermore, the up-regulation of SDF-1 was lower during the acute phase in the fracture-only group, in which the oxygen concentration was well preserved before surgery. This differential increase in SDF-1 expression between the two groups suggested that this molecule might be a key regulator involved in accelerating bone repair.

We observed that SDF-1 promoted the migration of MSCs in vitro in a dose-dependent manner, as based on in vitro and in vivo chemotactic assays. We also found that the BrdU-labeled mouse BMSCs that were injected intravenously could be recruited to the damaged bone. In addition, AMD3100, an antagonist of CXCR4, could inhibit this migration. These results strongly supported that SDF-1 was an essential molecule for the migration of MSCs to sites of bone repair in vivo. Recently, studies have shown that the recruitment of autologous stem cells may be substantially enhanced with localized release of stem cell chemokines [35]. It has been previously mentioned that the SDF-1α/CXCR4 axis might be involved in recruitment of expanded MSCs to damaged tissues [36]. Enhanced recruitment of autologous stem cells could improve the tissue responses and jumpstart stem cell participation in healing [37]. And our research has proven that SDF-1α is a promising candidate for in situ recruitment in bone regeneration [38]. However, the effects of SDF-1 in accelerated bone repair have not been shown. In our research, we demonstrated that the intravenously transplanted MSCs migrated to the site of bone repair in the TBI/fracture model. The mobilization of BrdU-positive cells was observed around the damaged bone, and AMD3100 could decrease the number of migrated cells. These results supported the idea that the SDF-1/CXCR4 axis was involved in the migration of cells to the sites of bone repair. Moreover, the percentage of BrdU-positive chondrocytes in the endochondral callus was 27.6%, demonstrating that the migrated cells had differentiated into chondrocytes. These results indicated that the migrated cells were mesenchymal cells and actually participated in endochondral bone formation. To our knowledge, this study is the first to show the effect of SDF-1 on accelerated bone repair in vivo. Another findings was that there was a significant correlation between the decreased volume of newly formed bone and the blockade of SDF-1 or CXCR4. The loss-of-function studies revealed that treatment with an anti–SDF-1 neutralizing antibody or AMD3100 remarkably decreased the area of new bone formation.

Taken together, these data indicated that the SDF-1/CXCR4 axis may play a key role in accelerated bone healing and contribute to endochondral bone repair. Further studies are needed to clarify the regulatory mechanism of SDF-1 expression in bone repair. Furthermore, we hope that the therapeutic use of SDF-1 to achieve successful bone repair will be feasible in the near future.

### Conclusion

The SDF-1/CXCR4 axis plays a crucial role in accelerated bone healing and contributes to endochondral bone repair in a TBI/fracture model. This study lays the foundation for the use SDF-1 to promote bone fracture healing in clinic setting.
